# The Conditional Protective Role of Psychological Resilience: School Bullying Climate, Emotional Regulation Difficulties, and Mobile Phone Dependence Among Left-Behind Children

**DOI:** 10.3390/bs16081261

**Published:** 2026-07-23

**Authors:** Chang Wei, Weibin Zhao, Chengfu Yu

**Affiliations:** 1 School of Arts and Sciences, Guangzhou Maritime University, Guangzhou 510725, China; 2 Department of Scientific Research, Guangzhou Maritime University, Guangzhou 510725, China; 3 School of Education, Guangzhou University, Guangzhou 510006, China

**Keywords:** left-behind children, school bullying climate, emotional regulation difficulties, mobile phone dependence, psychological resilience

## Abstract

School bullying climate is a known risk factor for adolescent mobile phone dependence, yet the mechanisms linking these two constructs and the boundary conditions of protective factors remain underexplored. The present study aimed to examine the mediating role of emotional regulation difficulties in the relationship between school bullying climate and mobile phone dependence among left-behind children, as well as the moderating role of psychological resilience in this mediating pathway. The final analytical sample consisted of 300 left-behind children who completed two waves of surveys separated by a six-month interval. Participants completed the School Bullying Climate Scale, the Brief Difficulties in Emotion Regulation Scale, the Mobile Phone Dependence Scale, and the Psychological Resilience Scale. Data were analyzed using the PROCESS macro for SPSS (Model 4 for mediation and Model 58 for moderated mediation), controlling for age, gender, and baseline mobile phone dependence. School bullying climate was significantly positively correlated with mobile phone dependence among left-behind children six months later (*r* = 0.14, *p* < 0.05), and emotional regulation difficulties mediated this relationship (indirect effect = 0.03, 95% CI = [0.001, 0.077]). Psychological resilience significantly moderated the path from school bullying climate to emotional regulation difficulties (*b* = 0.19, *p* < 0.05). Specifically, for left-behind children with high psychological resilience, the relationship between school bullying climate and emotional regulation difficulties was significant (*b* = 0.29, *p* < 0.01); for those with low psychological resilience, this relationship was not significant (*b* = 0.02, *p* > 0.05). Notably, psychological resilience played a conditional moderating role: only under a low school bullying climate is high psychological resilience associated with lower emotional regulation difficulties in left-behind children.

## 1. Introduction

According to the 5th National Survey Report on Internet Usage among Chinese Minors (China Internet Network Information Center, 2023), the number of underage internet users in China reached 193 million in 2022. The proportion of underage internet users accessing the internet via mobile phones reached 91.3%. Mobile phone dependence (also known as problematic mobile phone use or mobile phone addiction) is a behavioral addiction characterized by excessive or inappropriate use of mobile phones and psychological dependence on them, which in turn affects individuals’ physical and mental health as well as their social functioning (Zuo & Lu, 2020). Previous studies have found that mobile phone dependence not only leads to physiological problems such as poor sleep quality (Kumar et al., 2025), but is also closely related to mental health problems such as anxiety and depression (Augner et al., 2023; Carter et al., 2024). Left-behind children refer to children aged 0–17 who are unable to live with both of their parents due to one or both parents working or living outside their hometown for half a year or more (Lv et al., 2024). Previous studies have found that due to the lack of effective parental supervision, mobile phone dependence is relatively common among left-behind children, and their level of mobile phone dependence is significantly higher than that of children who are not left-behind (Xia et al., 2020). Therefore, there is an urgent need to explore the risk and protective factors of mobile phone dependence among left-behind children.

School bullying climate and bullying victimization are distinct but interrelated constructs: the former reflects students’ perceptions of the overall bullying environment at school, while the latter refers to individuals’ direct experiences of being bullied. According to the ecological systems theory (Bronfenbrenner, 1979), individual behavior and psychological adaptation are shaped not only by direct personal experiences (e.g., bullying victimization) but also by the characteristics of the broader environment in which individuals are embedded (e.g., school bullying climate). Moreover, empirical research has demonstrated a significant association between school climate and bullying victimization among left-behind children: students who perceive their school climate more negatively are more likely to report being victimized, and school climate has been found to be significantly negatively associated with various forms of bullying victimization (He et al., 2022). Therefore, findings from research on bullying victimization can provide important theoretical reference for understanding the mechanisms through which school bullying climate operates.

Previous research has confirmed that there is a close relationship between school climate and the emotional and behavioral problems of left-behind children (Yu, 2017). Based on the ecological systems theory (Bronfenbrenner, 1979), the school environment (e.g., school bullying climate) constitutes an important microsystem in the developmental process of left-behind children, exerting a significant influence on their behavioral development (e.g., mobile phone dependence). Consistent with this theory, existing research has confirmed a significant positive correlation between bullying and mobile phone dependence in adolescents. For example, in a longitudinal study of 737 adolescents, Li et al. (2025) found that bullying victimization significantly and positively predicted mobile phone dependence.

According to the general strain theory (Agnew, 1992), school bullying climate acts as a significant source of stress for left-behind children, causing them great psychological pressure, which in turn leads to emotional regulation difficulties. As a result, they may resort to mobile phone dependence as a means to escape reality and alleviate negative emotions. Previous studies have confirmed that school bullying is a key risk factor for emotional regulation difficulties (Vacca et al., 2023; S. Wang et al., 2025). Moreover, several studies have shown that emotional regulation difficulties, which are related to maladaptive behaviors in children and adolescents (Yu et al., 2025, 2026), are positively correlated with mobile phone dependence. For example, in a study of 720 Chinese adolescents, Fu et al. (2020) found that emotional regulation difficulties were positively associated with mobile phone dependence. In addition, prior studies have indicated that emotional regulation difficulties act as a mediator in the relationship between stress and problem behaviors among adolescents (Ao et al., 2025; Qiu et al., 2024). For example, in a sample of 476 senior high school students, Qiu et al. (2024) found that emotional regulation difficulties mediated the relationship between parental psychological control and suicidal ideation. Similarly, Ao et al. (2025) found that emotional regulation difficulties mediated the link between negative parenting styles and non-suicidal self-injury among adolescents.

The adverse effects of school bullying climate on individuals vary from one person to another, with psychological resilience being identified as one of the key factors contributing to these differences (Lu, 2025). Prior research has established that psychological resilience is a key protective factor against mobile phone dependence among adolescents (Geng et al., 2025). Psychological resilience refers to a dynamic process of positive adaptation by an individual in the face of adversity (Luthar et al., 2000). Based on the protective model of resilience (Fergus & Zimmerman, 2005), psychological resilience serves as a key protective factor that can moderate the relationship between risk factors and maladaptive outcomes. In the first stage, psychological resilience buffers the effect of school bullying (external risk) on emotional regulation difficulties (maladaptive outcome); in the second stage, psychological resilience further buffers the effect of emotional regulation difficulties (internal vulnerability risk factor) on mobile phone dependence (maladaptive outcome). Previous studies have confirmed that psychological resilience can weaken the negative impact of stress on adolescents’ maladaptive outcomes (Lu, 2025; Wei et al., 2022). For example, using a sample of 620 junior high school students, Lu (Lu, 2025) found that psychological resilience exerted a significant moderating effect on the association between bullying victimization and adolescent depression, and could effectively buffer the adverse effect of bullying victimization on depression.

**Figure 1:**
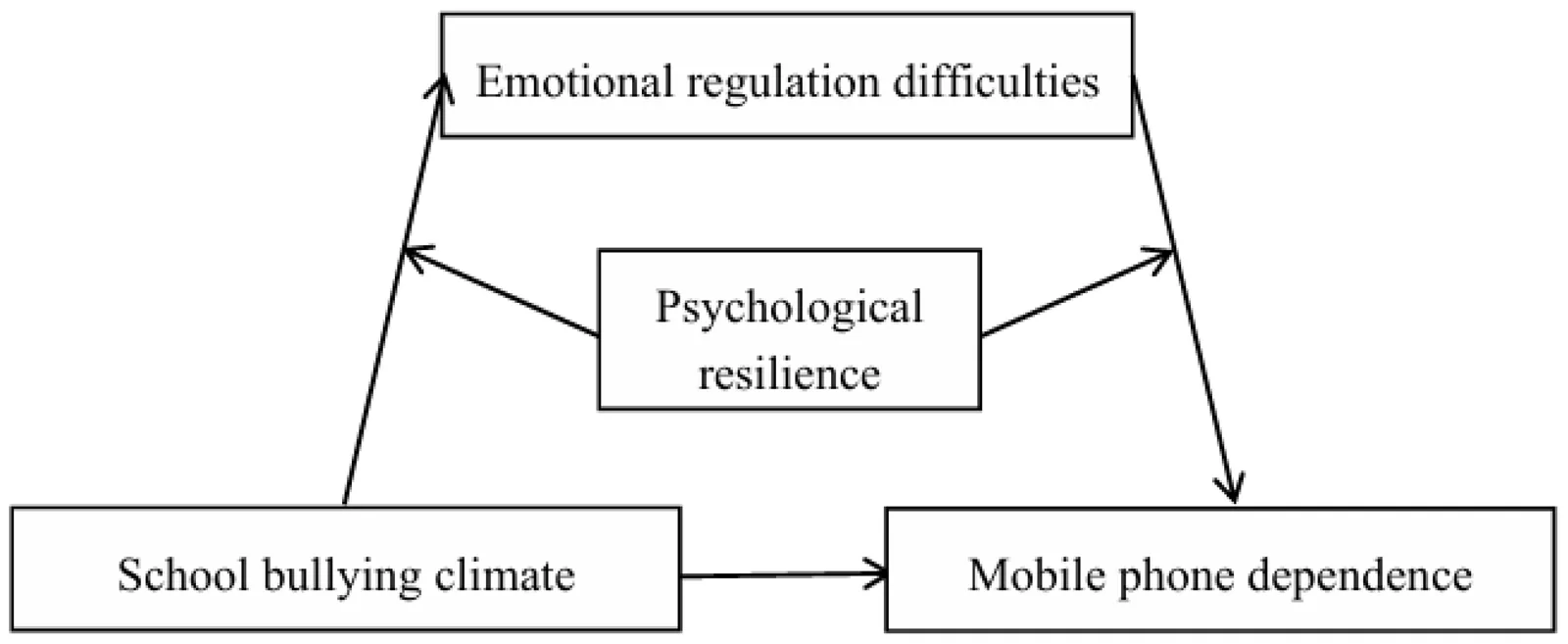
The proposed model.

Drawing on survey data from Chinese left-behind children, this study aimed to examine the mediating role of emotional regulation difficulties in the relationship between school bullying climate and mobile phone dependence, as well as the moderating role of psychological resilience in this mediating pathway (see Figure 1). Specifically, the following hypotheses were tested:

**Hypothesis** **1.** *School bullying climate is positively associated with mobile phone dependence among left-behind children six months later.*

**Hypothesis** **2.** *Emotional regulation difficulties mediate the prospective relationship between school bullying climate and mobile phone dependence.*

**Hypothesis** **3.** *Psychological resilience moderates the relationship between school bullying climate and emotional regulation difficulties. Similarly, psychological resilience moderates the relationship between emotional regulation difficulties and mobile phone dependence.*

## 2. Materials and Methods

### 2.1. Study Design and Sample

This study adopted a two-wave longitudinal design with a six-month interval. Using a cluster sampling approach, it recruited left-behind children from two junior high schools located in central China. Among the 340 left-behind children who participated in the first-wave survey, 323 completed the second-wave follow-up, with an overall attrition rate of 5.0%. Among these 323 respondents, we further excluded 23 participants with incomplete item responses, yielding a final analytical sample of 300 children. Comparisons of key variables between completers and dropouts showed no significant differences. We only used complete two-wave data for all statistical analyses. Among them, boys accounted for 51.0% and girls for 49.0%; 55.0% were in Grade 7 and 45.0% in Grade 8; 15.3% were only children and 84.7% had siblings; 81.7%% were single-left-behind children (with one parent migrating for work), and 18.3 were double-left-behind children (with both parents migrating). The age of the left-behind children ranged from 11 to 15 years, with a mean age of 13.30 years at the first survey.

### 2.2. Ethics Approval and Procedure

The study obtained ethical approval from the Academic Ethics Review Board of the university with which the author is affiliated. Written informed consent was obtained from parents, and assent was also secured from the left-behind children.

Inclusion Criteria: Written informed consent obtained from parents or legal guardians, along with verbal assent from the left-behind children themselves. Children met the official definition of “left-behind children”: minors aged 0–17 years who could not live with their parents because one or both parents had been working or residing in other locations for at least six consecutive months. Exclusion Criteria: Children who declined to participate in the questionnaire survey. Children who did not meet the left-behind definition. Students who were not currently enrolled in or attending school at the time of data collection.

Data were collected through direct paper-and-pencil surveys administered on-site in classroom settings, and questionnaires were collected immediately upon completion.

### 2.3. Measures

#### 2.3.1. School Bullying Climate

School bullying climate was measured using the School Bullying Subscale of the Delaware School Climate Scale (Bear et al., 2016), whose Chinese version was revised by Su et al. (2021). This Scale includes 4 items (such as “Students worry about being bullied by others”). Each item is scored on a 4-point scale (1 = strongly disagree to 4 = strongly agree). Mean scores were used for analysis, with higher scores indicating a higher level of school bullying climate perceived by the students. Cronbach’s alpha in this study was 0.79.

#### 2.3.2. Emotional Regulation Difficulties

Emotional regulation difficulties were assessed using the Chinese version of the Brief Difficulties in Emotion Regulation Scale (G. Wang et al., 2021), which was adapted from the original Brief Difficulties in Emotion Regulation Scale (Bjureberg et al., 2016). This scale includes 16 items (such as “My feelings confuse me”). Each item is rated on a 5-point scale (1 = never to 5 = always). Mean scores were used for analysis, with higher scores indicating a higher level of emotional regulation difficulties. Cronbach’s alpha in this study was 0.94.

#### 2.3.3. Psychological Resilience

Psychological resilience was assessed using the Chinese Version of the Resilience Scale (Tian, 2021), which was adapted from the Resilience Scale (Smith et al., 2008). This scale includes 6 items (such as “I can recover from stressful events in a short period of time”). Each item is rated on a 5-point scale (1 = strongly disagree to 5 = strongly agree). Mean scores were used for analysis, with higher scores indicating a higher level of psychological resilience. Cronbach’s alpha in this study was 0.72.

#### 2.3.4. Mobile Phone Dependence

Mobile phone dependence was assessed using the Chinese Version of the Smartphone Dependence Scale (Xiang et al., 2019), which was adapted from the Smartphone Dependence Scale (Kwon et al., 2013). This scale includes 10 items (such as “Unable to tolerate being without a smartphone”). Each item is rated on a 6-point scale (1 = strongly disagree to 6 = strongly agree). Mean scores were used for analysis, with higher scores indicating a higher level of mobile phone dependence. Cronbach’s alpha was 0.88 at Wave 1 and 0.94 at Wave 2.

### 2.4. Statistical Analyses

To analyze the data, we utilized SPSS 27.0 to generate descriptive statistics. We employed Model 4 of the PROCESS macro to examine whether emotional regulation difficulties mediated the relationship between school bullying climate and mobile phone dependence. Additionally, we employed Model 58 of the PROCESS macro to examine the moderating effect of psychological resilience on both the direct and indirect associations between school bullying climate and mobile phone dependence via emotional regulation difficulties. In addition, gender, age, and baseline mobile phone dependence were included as covariates in the statistical models to control for their potential effects.

## 3. Results

### 3.1. Common Method Bias Test

To test for common method bias, Harman’s single-factor test was conducted by including all items of self-report scales measured at Wave 1 in an unrotated exploratory factor analysis. The results revealed that the first common factor accounted for 27.90% of the total variance, which was below the 40% cutoff criterion, indicating no serious common method bias in this study.

### 3.2. Preliminary Analyses

The descriptive statistics, including means and standard deviations, as well as the correlation coefficients for all variables, are illustrated in Table 1. School bullying climate at Wave 1 was significantly positively related to mobile phone dependence at Wave 1 and Wave 2. School bullying climate at Wave 1 was significantly positively related to emotional regulation difficulties at Wave 1; emotional regulation difficulties at Wave 1 were significantly positively related to mobile phone dependence at Wave 1 and Wave 2. Additionally, psychological resilience at Wave 1 was significantly negatively related to mobile phone dependence at Wave 1 and Wave 2.

### 3.3. Mediation Effect of Emotional Regulation Difficulties

**Figure 2:**
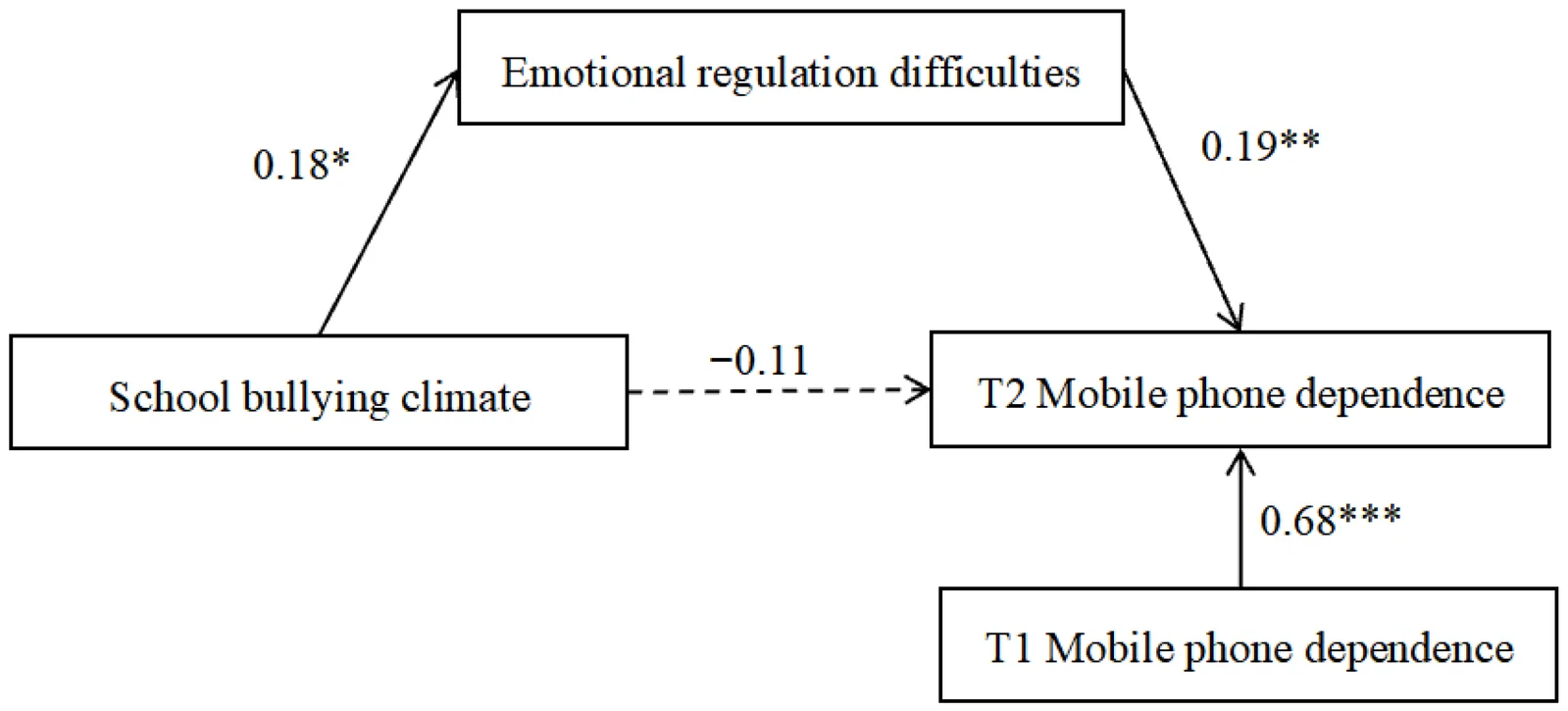
Model of the mediating role of emotional regulation difficulties in the association between school bullying climate and mobile phone dependence. * *p* < 0.05. ** *p* < 0.01. *** *p* < 0.001.

The results of the mediation model are depicted in Figure 2. After controlling for age, gender, and mobile phone dependence at Wave 1, it was found that school bullying climate at Wave 1 could positively predict emotional regulation difficulties at Wave 1 (*b* = 0.18, *SE* = 0.07, *β* = 0.14, *p* < 0.05), and emotional regulation difficulties at Wave 1 could positively predict mobile phone dependence at Wave 2 (*b* = 0.19, *SE* = 0.07, *β* = 0.14, *p* < 0.01. The bias-corrected percentile bootstrap (5000 resamples) method showed a significant mediating effect of emotional regulation difficulties in the relationship between school bullying climate and mobile phone dependence (indirect effect = 0.03, *SE* = 0.02, 95% CI = [0.001, 0.077]), with a completely standardized indirect effect of 0.02.

### 3.4. Moderation Effect of Psychological Resilience

**Figure 3:**
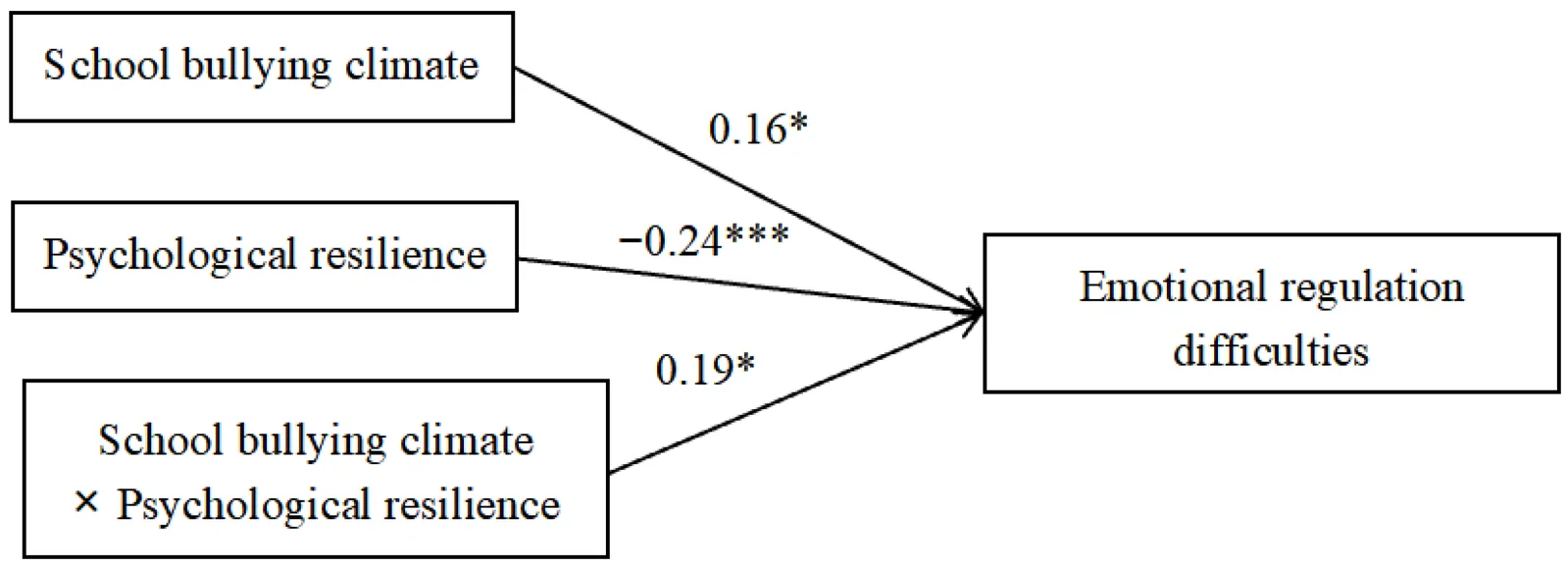
The moderating role of psychological resilience on the relationship between school bullying climate and emotional regulation difficulties. * *p* < 0.05. *** *p* < 0.001.

All continuous predictor variables were mean-centered prior to the construction of interaction terms to reduce multicollinearity. We ran bias-corrected bootstrapping with 5000 resamples to test indirect effects. After controlling for age, gender, and mobile phone dependence at Wave 1, psychological resilience significantly moderated the path from school bullying climate to emotional regulation difficulties (*b* = 0.19, *SE* = 0.09, *β* = 0.11, *p* < 0.05, Δ*R*^2^ = 0.01, *f*^2^ = 0.015), supporting the hypothesized moderating role of psychological resilience on the first-stage path. The results are depicted in Figure 3. However, psychological resilience did not significantly moderate the path from emotional regulation difficulties to mobile phone dependence (*b* = −0.12, *SE* = 0.07, *β* = −0.08, *p* > 0.05, Δ*R*^2^ = 0.01, *f*^2^ = 0.011), failing to support the hypothesized moderating role of resilience on the second-stage path.

**Figure 4:**
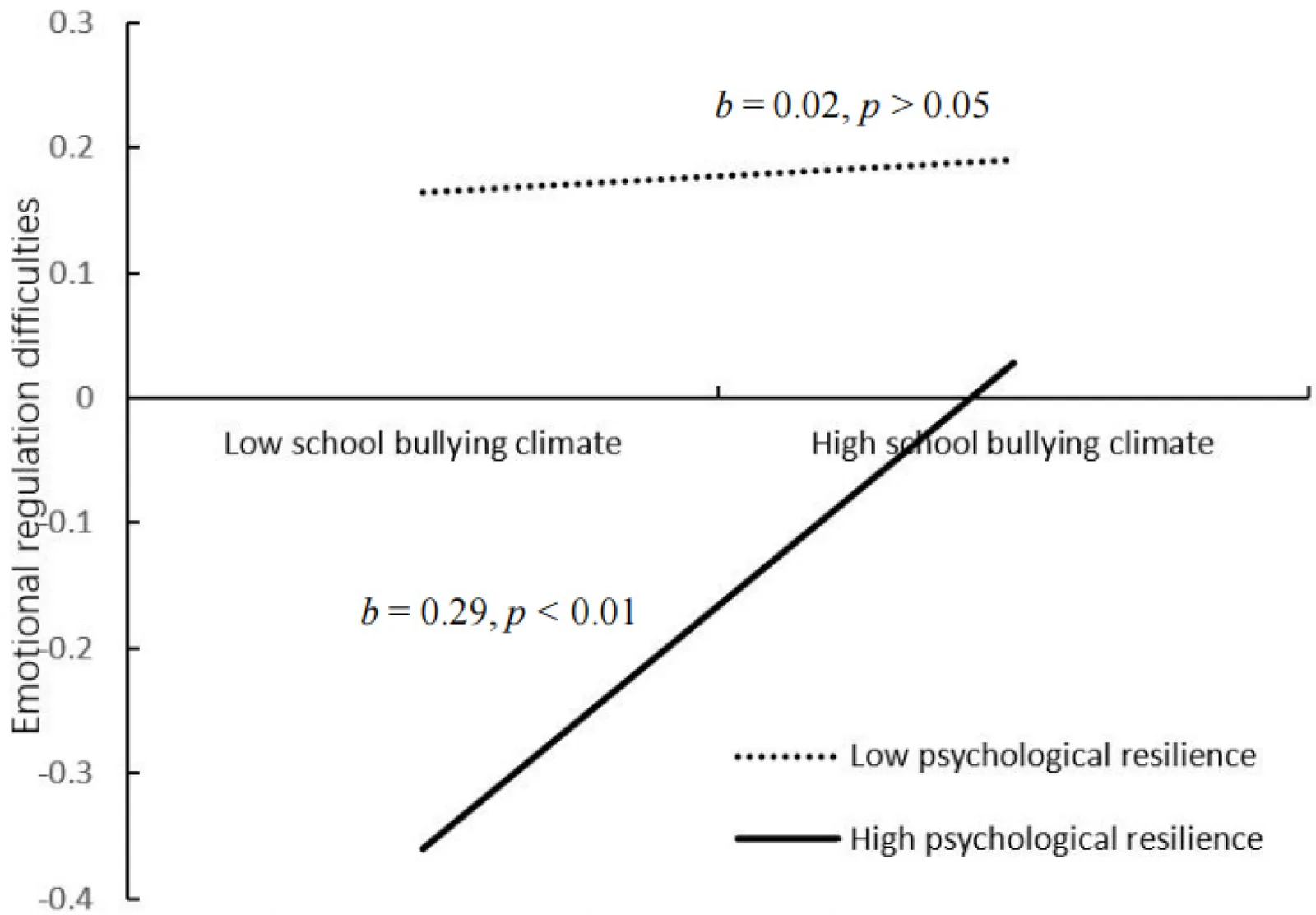
Interactive effect of school bullying climate and psychological resilience on emotional regulation difficulties.

In addition, we examined conditional indirect effects at two levels of psychological resilience (−1 SD and +1 SD). The results showed that the indirect effect of school bullying climate on mobile phone dependence via emotional regulation difficulties was statistically non-significant at both high resilience (indirect effect = 0.02, *SE* = 0.03, 95% CI = [−0.03, 0.08]) and low resilience (indirect effect = 0.005, *SE* = 0.03, 95% CI = [−0.06, 0.07]). This indicates that although psychological resilience significantly moderated the first-stage path (school bullying climate → emotional regulation difficulties), this moderating effect failed to propagate through the full indirect pathway. To better understand the significant local moderation, we conducted simple slopes tests. As depicted in Figure 4, when left-behind children showed higher psychological resilience, the relation between school bullying climate and emotional regulation difficulties was significant (*b* = 0.29, *SE* = 0.09, *p* < 0.01). However, when left-behind children showed lower psychological resilience, the relation was not significant (*b* = 0.02, *SE* = 0.10, *p* > 0.05). The overall model accounted for 22% of the variance in emotional regulation difficulties (*R*^2^ = 0.22) and 47% of the variance in mobile phone dependence (*R*^2^ = 0.47).

## 4. Discussion

This study explored the association between school bullying climate and mobile phone dependence among left-behind children and further examined the mediating role of emotional regulation difficulties and the moderating role of psychological resilience. The results show a significant positive correlation between school bullying climate and mobile phone dependence, with emotional regulation difficulties acting as a mediator. It is noteworthy that the protective effect of psychological resilience is conditional: only under a low school bullying climate can high psychological resilience reduce emotional regulation difficulties among left-behind children.

Consistent with Hypothesis 1, the present study found a significant positive correlation between school bullying climate and mobile phone dependence among left-behind children six months later, suggesting that school bullying climate is associated with an increased risk of mobile phone dependence in left-behind children. This finding supports previous research and further extends the evidence on the association between school climate and internalizing and externalizing problem behaviors among left-behind children (Yu, 2017). According to the ecological systems theory (Bronfenbrenner, 1979), school bullying climate is an important environmental factor within the microsystem of child development. When left-behind children are exposed to a bullying climate, they may use mobile phones as a coping strategy to escape real-life stress, thereby increasing the risk of mobile phone dependence. Therefore, left-behind children exposed to a high level of school bullying climate are more likely to exhibit mobile phone dependence behaviors.

Consistent with Hypothesis 2, this study found that emotional regulation difficulties served as a significant mediator in the relationship between school bullying climate and mobile phone dependence, indicating that emotional regulation difficulties represent a key linking mechanism. The findings suggest a pathway from school bullying climate to emotional regulation difficulties and then to mobile phone dependence, providing empirical evidence for the processes through which school bullying climate is associated with mobile phone dependence among left-behind children. This finding is in line with previous studies (Ao et al., 2025; Qiu et al., 2024) and further confirms the mediating role of emotional regulation difficulties between stress and problem behaviors in children and adolescents. It also supports the general strain theory (Agnew, 1992). School bullying climate may lead to negative emotions among left-behind children. If these negative emotions are not effectively regulated, they can further exacerbate maladaptive behaviors, such as mobile phone dependence.

This study found that psychological resilience significantly moderated the relationship between school bullying climate and emotional regulation difficulties. Specifically, at high levels of psychological resilience, school bullying climate significantly positively predicted emotional regulation difficulties, whereas at low levels of psychological resilience, this predictive effect was non-significant; regardless of the level of school bullying climate, left-behind children with low psychological resilience consistently showed high levels of emotional regulation difficulties. This pattern suggests that the protective role of psychological resilience manifests primarily as significantly reducing emotional regulation difficulties among left-behind children under low-stress conditions.

High psychological resilience appeared to amplify the effect of school bullying climate on emotional regulation difficulties, which does not mean that psychological resilience itself exerts negative effects. One possible explanation is that left-behind children with low psychological resilience have approached ceiling levels in emotional regulation difficulties, leading to restricted variance, making the incremental effect of school bullying climate difficult to detect. Under a high school bullying climate, continuous coping by individuals with high psychological resilience rapidly depletes their psychological resilience resources (Hobfoll, 2002), causing the protective buffering mechanism to fail, and emotional regulation difficulties increase sharply and gradually converge toward the levels of left-behind children with low psychological resilience. Another possible explanation relates to the developmental context of left-behind children. Compared with their non-left-behind peers, left-behind children lack long-term parental care and companionship, and endure persistent stress stemming from inadequate family support. This keeps their psychological resilience resources under sustained depletion, which may weaken the protective effect of resilience when coping with a school bullying climate.

These findings are not inconsistent with previous research proposing that “psychological resilience serves as a protective factor mitigating the negative impact of stress” (Wei et al., 2022), but rather further supplement the boundary conditions of this protective effect: under low-stress environments, left-behind children with high psychological resilience demonstrate superior emotional regulation compared to those with low psychological resilience; yet under high-intensity environmental adversity, resilience resources are heavily depleted, their buffering function dissipates, and the advantage of high psychological resilience is significantly attenuated. This suggests that psychological resilience does not operate as an unconditionally effective protective factor, but rather functions within specific contextual limits.

This study has several important implications for prevention and intervention strategies. First, given that school bullying climate is an important risk factor for mobile phone dependence, it is necessary to take effective measures to improve this situation. For example, schools can strengthen class ethos and cultural construction to create a more positive and harmonious school atmosphere (Bao et al., 2025), thereby effectively reducing school bullying climate. Second, this study reveals that emotional regulation difficulties mediate the relationship between school bullying climate and mobile phone dependence, highlighting the importance of focusing on the cultivation of emotional regulation skills among left-behind children in intervention measures. For example, schools can offer specialized courses on emotional management and provide professional psychological counseling services to enhance their emotional regulation abilities. Lastly, the results indicate that the protective effect of psychological resilience is conditional: only under a low school bullying climate is high psychological resilience associated with lower emotional regulation difficulties in left-behind children. Therefore, school interventions should adopt a differentiated approach. In schools with a low bullying climate, interventions may focus on enhancing left-behind children’s psychological resilience through social skills and emotional regulation training (González Moreno & Molero Jurado, 2024; Zhang & Xu, 2022), thereby leveraging its protective advantages and reducing emotional regulation difficulties. However, in schools with a high bullying climate, simply enhancing psychological resilience may be insufficient to exert its protective function. Instead, schools should prioritize addressing and reducing the school bullying climate, thereby creating the prerequisite conditions for psychological resilience to serve its buffering role.

This study had several limitations. First, this study adopted a single-source self-report design, which raises the risk of common method bias. While the two-wave longitudinal design with a six-month interval can mitigate this risk to a certain degree, common method bias is still an unavoidable limitation of the current research. To improve measurement objectivity and the validity of research conclusions, future research can adopt multi-informant evaluation data, including reports from caregivers, homeroom teachers and peers. Second, this study only investigated the mediating role of emotional regulation difficulties in the relationship between school bullying climate and mobile phone dependence. Future research could further examine the mediating role of other variables, such as maladaptive cognitions (R. Liu et al., 2019), to gain a more comprehensive understanding of this relationship. Third, this study only examined the moderating role of psychological resilience in the relationship between school bullying climate and emotional regulation difficulties, as well as between emotional regulation difficulties and mobile phone dependence. It did not explore whether other individual-level variables (e.g., self-compassion (Q. Liu et al., 2020)) might also exert buffering effects at different stages of this process. Future research could incorporate more individual-level variables to gain a deeper understanding of the relationship between school bullying climate and mobile phone dependence among left-behind children. Fourth, although the two-wave longitudinal design with a six-month interval allowed us to examine the temporal precedence of the variables, it could not fully establish causal relationships among them. Future research could employ multi-wave longitudinal designs to further strengthen causal inference. Fifth, given that the sample consisted entirely of left-behind children from a Chinese cultural background, the cross-cultural generalizability of our findings remains unclear. Future studies could conduct investigations in diverse cultural groups to test whether the conclusions hold across different cultural contexts.

## 5. Conclusions

The present study found a significant positive correlation between school bullying climate and mobile phone dependence among left-behind children, with emotional regulation difficulties mediating this relationship. Notably, psychological resilience played a conditional moderating role: only under a low school bullying climate is high psychological resilience associated with lower emotional regulation difficulties in left-behind children.

## Figures and Tables

**Table 1:** Descriptive statistics and correlations for all variables.

Variable	1	2	3	4	5	6	7
1. Gender	1.00						
2. Age	0.05	1.00					
3. SBC	0.07	0.12 *	1.00				
4. ERD	−0.05	0.10	0.24 ***	1.00			
5. PR	0.05	−0.05	−0.06	−0.29 ***	1.00		
6. T1 MPD	−0.05	0.04	0.29 ***	0.37 ***	−0.24 ***	1.00	
7. T2 MPD	−0.11	−0.02	0.14 *	0.35 ***	−0.26 ***	0.66 ***	1.00
Mean	0.51	13.30	1.94	2.03	3.27	2.61	2.37
SD	0.50	0.77	0.67	0.87	0.71	1.11	1.22

Note: Gender was dummy coded as 1 = male, 0 = female. SBC = school bullying climate; ERD = emotional regulation difficulties; PR = psychological resilience; MPD = Mobile phone dependence. * *p* < 0.05. *** *p* < 0.001.

## Data Availability

The data presented in this study are available from the author upon reasonable request (the data are not publicly available due to privacy or ethical restrictions).

## Abbreviations

The following abbreviations are used in this manuscript:

SBC	School bullying climate
ERD	Emotional regulation difficulties
PR	Psychological resilience
MPD	Mobile phone dependence
LBC	Left-behind children
